# Cortical Somatosensory Reorganization in Children with Spastic Cerebral Palsy: A Multimodal Neuroimaging Study

**DOI:** 10.3389/fnhum.2014.00725

**Published:** 2014-09-12

**Authors:** Christos Papadelis, Banu Ahtam, Maria Nazarova, Donna Nimec, Brian Snyder, Patricia Ellen Grant, Yoshio Okada

**Affiliations:** ^1^Fetal-Neonatal Neuroimaging and Developmental Science Center, Boston Children’s Hospital, Harvard Medical School, Boston, MA, USA; ^2^Division of Newborn Medicine, Department of Medicine, Boston Children’s Hospital, Harvard Medical School, Boston, MA, USA; ^3^Department of Neurorehabilitation and Physiotherapy, Research Center of Neurology, Moscow, Russia; ^4^Centre for Cognition and Decision Making, Faculty of Psychology, Higher School of Economics, Moscow, Russia; ^5^Department of Orthopaedic Surgery, Boston Children’s Hospital, Harvard Medical School, Boston, MA, USA; ^6^Department of Radiology, Boston Children’s Hospital, Harvard Medical School, Boston, MA, USA

**Keywords:** cerebral palsy, cortical reorganization, somatosensory processing, magnetoencephalography, MR tractography, resting-state functional MRI

## Abstract

Although cerebral palsy (CP) is among the most common causes of physical disability in early childhood, we know little about the functional and structural changes of this disorder in the developing brain. Here, we investigated with three different neuroimaging modalities [magnetoencephalography (MEG), diffusion tensor imaging (DTI), and resting-state fMRI] whether spastic CP is associated with functional and anatomical abnormalities in the sensorimotor network. Ten children participated in the study: four with diplegic CP (DCP), three with hemiplegic CP (HCP), and three typically developing (TD) children. Somatosensory (SS)-evoked fields (SEFs) were recorded in response to pneumatic stimuli applied to digits D1, D3, and D5 of both hands. Several parameters of water diffusion were calculated from DTI between the thalamus and the pre-central and post-central gyri in both hemispheres. The sensorimotor resting-state networks (RSNs) were examined by using an independent component analysis method. Tactile stimulation of the fingers elicited the first prominent cortical response at ~50 ms, in all except one child, localized over the primary SS cortex (S1). In five CP children, abnormal somatotopic organization was observed in the affected (or more affected) hemisphere. Euclidean distances were markedly different between the two hemispheres in the HCP children, and between DCP and TD children for both hemispheres. DTI analysis revealed decreased fractional anisotropy and increased apparent diffusion coefficient for the thalamocortical pathways in the more affected compared to less affected hemisphere in CP children. Resting-state functional MRI results indicated absent and/or abnormal sensorimotor RSNs for children with HCP and DCP consistent with the severity and location of their lesions. Our findings suggest an abnormal SS processing mechanism in the sensorimotor network of children with CP possibly as a result of diminished thalamocortical projections.

## Introduction

Cerebral palsy (CP) is a well-recognized group of motor and postural neurodevelopmental disorders beginning in early childhood and persisting through the lifespan. CP causes serious motor impairments often accompanied by disturbances of sensation and perception (Bax et al., 2005) and is the most common cause of physical disability in early childhood (Krageloh-Mann and Cans, 2009). Almost 3.6 out of every 1000 children born in the US suffer from CP and the prevalence of the disorder is on the rise worldwide (Yeargin-Allsopp et al., 2008). CP involves axonal and neuronal loss in cerebral white and gray matter, reduction in thalamocortical connections (Hoon et al., 2002, 2009; Thomas et al., 2005; Nagae et al., 2007), and comparable loss in subcortical structures (Volpe, 2009). Depending on the location, extent, and timing of the insult, clinical symptoms vary largely.

Spastic CP (SCP) is the most common form of CP that frequently alters the normal development of the somatosensory (SS) system. SCP is usually presented with increased muscle tone, hyperreflexia, and persistence of primitive reflexes (Tomlin, 1995). Aside from motor and postural impairments, children with SCP frequently experience sensory deficits such as altered tactile, proprioceptive, kinesthetic, and pain awareness (Van Heest et al., 1993; Cooper et al., 1995; Krumlinde-Sundholm and Eliasson, 2002; Sanger and Kukke, 2007; Wingert et al., 2009; Riquelme et al., 2011). Recent functional neuroimaging studies have reported altered SS processing in SCP individuals as measured by SS-evoked potentials or fields in terms of amplitude, morphology, frequency power, or somatotopy (Riquelme and Montoya, 2010; Kurz and Wilson, 2011; Teflioudi et al., 2011; Guo et al., 2012; Nevalainen et al., 2012; Pihko et al., 2014; Riquelme et al., 2014). In a recent magnetoencephalography (MEG) study, Pihko et al. (2014) found that suppression and rebound of alpha and beta cortical activity to contralateral stimulation were smaller in the lesioned compared to the intact hemisphere in hemiplegic CP (HCP) children, while they did not find any difference between the hemispheres of typically developing (TD) children. A functional MRI study, which investigated tactile shape and grating discrimination, found decreased cortical activity in the parietal and frontal cortical SS regions of SCP compared to TD children (Wingert et al., 2010).

Anatomical neuroimaging studies using diffusion tensor imaging (DTI) have provided evidence of significant alterations in white matter fibers connecting to sensory cortex. These studies suggest that CP injuries might be reflective of disruption of sensory as well as motor connections. They also provide evidence of sensory and motor pathway involvement for the motor weakness in CP patients by showing that DTI measures reflect the degree of motor deficits (Hoon et al., 2002, 2009; Thomas et al., 2005; Trivedi et al., 2010). Two DTI studies observed more severe damage in the posterior white matter fibers connecting the thalamus to the sensory cortex than in the descending corticospinal tracts in children with periventricular leukomalacia (PVL) (Hoon et al., 2002) and SCP (Nagae et al., 2007), despite a history and clinical presentation consistent with motor tracts. Compared to TD children, children with CP have been reported to have decreased fractional anisotropy (FA) and increased apparent diffusion coefficient (ADC) values for the motor and sensory tracks (Trivedi et al., 2010); for the affected side of the corticospinal tract (Son et al., 2007; Yoshida et al., 2010); and for the tracks on the side ipsilateral to the periventricular lesion in corticospinal tract, corticobulbar tract, and superior thalamic radiation (Thomas et al., 2005).

Fundamental understanding of sensory function in CP children is extremely important, since SS input is an essential component of motor function, control, and development. Tactile inputs are used to localize and characterize the various qualities of touch, while cutaneous inputs contribute to proprioceptive information for coordinated motor actions (Dijkerman and de Haan, 2007). Both tactile and cutaneous inputs play an important role in the proprioceptive feedback for motor planning and execution (Wingert et al., 2008). Sensibility deficiencies have been correlated with diminished dexterity in the affected hand of CP children with spastic hemiplegia (Van Heest et al., 1993). It has been suggested that in CP there is a loss of coordinated messages from SS to motor areas (Burton et al., 2009), which may lead to deficits in motor coordination (Bax et al., 2005), fine and gross motor function (Himmelmann et al., 2006), and motor control (Ostensjo et al., 2004; Fowler and Goldberg, 2009). Deficits in the processing of SS information may also partially explain the tactile or motor deficits observed in this population (Wingert et al., 2008; Burton et al., 2009).

Somatosensory inputs are also important for the development of motor system; early learning in infants is driven largely by SS inputs. Moreover, the ability to process and utilize sensory information for motor planning control develops through the childhood (Riquelme and Montoya, 2010; Gordon et al., 2013). Thus, defective tactile/cutaneous feedback may worsen motor planning and performance (Gordon et al., 2013). CP children with even mild motor impairments have demonstrated more variable and redundant fingertip forces while adjusting to objects compared to TD, presumably, at least partly, due to imperfect proprioceptive feedback (Gordon et al., 2013). It is possible that there is also an opposite mechanism: deficits of spontaneous movements in CP infants (Prechtl, 1997; Prechtl et al., 1997; Hadders-Algra, 2004; Einspieler and Prechtl, 2005) may account for musculoskeletal tissue changes and thus contribute to aberrant sensory inputs to the brain (Coq et al., 2008). Understanding the pathophysiology mechanisms underlying the sensory impairments, specifically tactile, in CP children is essential to the design of effective therapeutic interventions.

In the present study, we investigated with three different neuroimaging modalities [pediatric MEG, DTI, and resting-state functional MRI (rs-fMRI)] whether SCP is associated with functional and anatomical abnormalities in the sensorimotor network. To our best knowledge, this is the first study that combines findings from multiple neuroimaging techniques for examining the anatomical and functional integrity of the SS and motor systems in children with SCP. Somatosensory-evoked fields (SEFs) in response to pneumatic stimulation of the fingertips were recorded with pediatric MEG and the underlying generators were localized by using minimum norm estimates (MNE) (Hamalainen and Ilmoniemi, 1994). MEG was used because it is able to elucidate the dynamic spatiotemporal characteristics of SS cortical activation (Lin et al., 2006a) with its excellent temporal and good spatial accuracy (Hämäläinen et al., 1993; Papadelis et al., 2009). So far, there are very few MEG studies reporting functional abnormalities in the SS cortex of CP children (Kurz and Wilson, 2011; Nevalainen et al., 2012; Pihko et al., 2014). With MEG, we tested our hypotheses that the SEFs will be abnormal and the primary cortical SS representation areas will present an altered somatotopy in the affected (or more affected) compared to the intact (or less affected) hemisphere of CP children, and compared to both hemispheres of TD children. The integrity of the thalamocortical tracts from thalamus to the pre-central and post-central gyri was examined in the same participants by using DTI. We expected a disruption in the thalamocortical tracts projecting from thalamus to the pre-central and post-central gyri in children with CP. The integrity of the sensorimotor resting-state network (RSN) functional architecture was also assessed by measuring spontaneous low-frequency fluctuations (<0.1 Hz) in the blood oxygen level-dependent (BOLD) signal by using an independent component analysis (ICA) method. We hypothesized that the cortical networks linked to areas within the SS and motor cortices would be either absent or at least abnormal in children with CP.

## Materials and Methods

### Participants

Ten right-handed children participated in the study: four children with diplegic CP (DCP) (two males and two females, mean age: 11.8 years, SD 5.4 years), three children with HCP (two males and one female, mean age: 12.6 years, SD 5.8 years), and three age-matched TD children (one male and two females, mean age: 11.8 years, SD 5.2 years). CP patients were recruited from the Cerebral Palsy Clinic at Boston Children’s Hospital (BCH), Harvard Medical School, according to the following inclusion criteria: (1) evaluation by a pediatric neurologist and diagnosis of DCP or HCP, (2) absence of any genetic syndrome diagnosis, (3) no history of trauma or brain operation, and (4) classified as high-functioning in level I or II at the Gross Motor Function Classification System (GMFCS) (Palisano et al., 1997). TD comparison children had no history of neurological disorder or brain injury. None of the participants were under psychoactive or myorelaxant medications during the experiment. Table 1 presents the demographic, clinical, and conventional MRI data for all participants. All participants were free of metallic objects or implant devices, and cooperative to understand and follow simple instructions. This study was approved by the local institutional review board and informed written consent was obtained from the parents of all participants.

**Table 1:** **Demographics and anatomical information**.

Participant	Age	Gender	GMFCS	Affected side	Brain MRI
TD 1	12 years	F	N/A	N/A	Normal
TD 2	17 years	F	N/A	N/A	Normal
TD 3	6.5 years	M	N/A	N/A	Normal
HCP 1	17 years	M	2	Right	Chronic encephalomalacia due to left middle cerebral artery infarct
HCP 2	15 years	F	2	Left	Perinatal right periventricular white matter injury
HCP 3	6 years	M	1	Right	Subtle gliosis in the left central semiovale extending to the corona radiata
DCP 1	12 years 9 months	F	2	Both (right more)	Slight asymmetry of the left lateral ventricle
DCP 2	12 years	F	2	Both (right more)	Bilateral periventricular white matter injury
DCP 3	16 years	M	1	Both (left more)	No gross MRI abnormality
DCP 4	4 years 9 months	M	2	Both (right more)	Periventricular leucomalacia (PVL)^a^

*^a^MRI data were excluded due to excessive motion artifact. PVL diagnosis was given based on previous acquired MRI not available to this study*.

### MEG recordings

SEFs were elicited by tactile stimulation of the D1, D3, and D5 (thumb, middle, and little finger) of both hands. The stimuli were delivered through thin elastic membranes (with a diameter of 1 cm), surrounded by a plastic outer shell, which were attached to the distal, volar parts of the three digits. Membranes were inflated by an air pressure pulse through a rigid plastic tube (Somatosensory Stimulus Generator, 4D NeuroImaging Inc., San Diego, CA, USA) tapping gently the skin at the tip of each digit. The pressure of the tactile stimulator rose to 0.10 bar overpressure in 10 ms. Tactile stimuli were delivered to the three digits in a pseudorandom order with an interstimulus interval (ISI) of 1.5 ± 0.5 s. Each finger received in total 180 stimuli in three runs. Each run lasted ~6 min. The measurements were carried out in the BabyMEG Facility at BCH (Waltham, MA, USA). MEG signals were recorded inside a single-layer magnetically shielded room (MSR) (Imedco, Hägendorf, Switzerland) with a 76-axial gradiometers device (BabySQUID Tristan Technologies, Inc., San Diego, CA, USA). The sensor array in BabySQUID consists of 74 active gradiometers (10 mm pickup coil diameter, 30 mm baseline, and 12–14 mm coil center-to-coil center spacing) with a gap between each pickup coil and scalp of ~7–10 mm. The sensor array is ellipsoidal in shape with a radius of curvature of 7.5 cm along the coronal section, 10 cm along the sagittal section (Okada et al., 2006), and a depth of ~2 cm resulting in a coverage area of ~265 cm^2^. The recording signal bandwidth was 0–341.33 Hz with a sampling rate of 1024 Hz. Vertical and horizontal electrooculograms (EOGs) and electrocardiogram (ECG) were simultaneously recorded using bipolar electrodes.

During the recordings, the children were comfortably lying down on the bed with the hemisphere contralateral to the stimulated hand placed on the headrest in such a way that the sensor array was covering the scalp area above the pre-central and post-central gyri. We focused our recordings on the contralateral hemisphere because previous evidence in CP children present that the SS representation remains in the hemisphere contralateral to the affected hand in contrast to the motor system (Gerloff et al., 2006; Staudt et al., 2006; Wilke and Staudt, 2009). The children kept their head still during the recordings with eyes open gazing at a fixation point on the wall. Prior to each run an anatomical coordinate system was defined by digitizing 15 points marked on the participant’s face. More details about the followed coregistration procedure can be found in Papadelis et al. (2013).

### MRI/DTI acquisition

MRI data were collected with a 3-T Siemens Tim Trio MR scanner at BCH. The data from one child with DCP (DCP4) were excluded due to excessive motion artifact. The scans were performed without sedation or medication, while the children were awake. The imaging protocol consisted of structural and diffusion-weighted sequences. The structural sequence was a T1-weighted high-resolution magnetization-prepared rapid-acquisition gradient-echo (MPRAGE) acquisition, which used volumetric echo-planar imaging (EPI) navigators for real time motion correction [voxel size (mm) = 1 × 1 × 1; field of view (FOV) = 19.2–22.0 cm; echo time (TE) = 1.74 ms; repetition time (TR) = 2520 ms; flip angle = 7°]. The diffusion sequence (prescribed axially) used echo-planar (EP) readouts [voxel size (mm) = 2.0 × 2.0 × 2.0; FOV = 11–12.8 cm; TE = 88 ms; TR = 8320–10934 ms; flip angle = 90°; 30 gradient diffusion directions at *b* = 1000 s/mm^2^; 10 acquisitions with *b* = 0 s/mm^2^].

### rs-fMRI recordings

The rs-fMRI data were collected with the same scanner at BCH after collecting the T1 and DTI sequences. The data from one child with DCP (DCP4) were excluded due to excessive motion artifact. The participants were asked to stay awake and keep their eyes open. The rs-fMRI sequence used EPI readouts with isotropic voxel sizes of 3 mm × 3 mm × 3 mm, whole brain coverage, TE = 30 ms, TR = 3 s, 47 axial slices, and 160 time series volumes.

### Analysis of event-related magnetic fields

MEG data analysis was performed by using Brainstorm (Tadel et al., 2011), which is documented and freely available for download online under the GNU general public license^1^. The recorded MEG signals were visually inspected for possible artifacts and filtered offline in the frequency band of 1–100 Hz. Trials contaminated by prominent eye movements, blinks, or muscular activity were rejected. The remaining trials were then averaged (approximately 60 trials for each stimulation site) for each separate run from −100 to 400 ms relative to the stimulus onset. Grand averages across all runs were estimated per stimulation site for each participant. There was no significant difference in the number of useful trials between stimulation sites or groups (*p* > 0.05). The peak latency of the first cortical response was visually determined from the grand average butterfly plot for each site and each participant.

### Source localization analysis

An MRI-derived surface model of each participant’s brain was initially estimated from the T1-weighted structural volumetric images by using FreeSurfer^2^. For the participant with no MRI, a template MRI from an age-matched child was used. The geometry of the gray–white matter surface was derived with an automatic segmentation algorithm to yield a triangulated model with approximately 270,000 vertices (Dale et al., 1999; Fischl et al., 1999, 2001). For computational purposes, the source space was obtained by decimating the original triangulation to a subset of vertices (~15,000 vertices) with an average of 5 mm distance between nearest dipoles. Each vertex represents a given source space point defined as an equilateral triangle in the tessellation of the cortical surface. To compute the forward model, the overlapping-sphere method was used for each participant that fits one local sphere for each sensor (Huang et al., 1999) and then derives the strength of a set of current dipoles located at the cortical surface. The distributed source model of the MEG signals was then estimated by using the MNE (Hamalainen and Ilmoniemi, 1994). MNE was selected because it does not require explicit *a priori* assumptions about the nature or number of source currents (Hamalainen and Ilmoniemi, 1994). It has been suggested to be the preferred method when analyzing multi-source SS-evoked activations compared to other inverse approaches (Lin et al., 2006a). Using distributed source analysis, the activation at each vertex was estimated at the peak of the first cortical response in the evoked fields obtained for each stimulus site and subject. During the computation of the inverse solution, we followed a previously described data analysis strategy (Hsiao et al., 2013): (i) the source orientations were constrained to be perpendicular to the cortical surface; (ii) a depth weighting algorithm was used to compensate for any bias affecting the superficial sources calculation (Lin et al., 2006b); and (iii) a regularization parameter, λ^2^ = 0.33 was used to minimize numerical instability to reduce the sensitivity of the MNE to noise and to effectively obtain a spatially smoothed solution (Hamalainen and Ilmoniemi, 1994). The noise covariance matrix was computed from empty MSR recordings, which always preceded the actual recording sessions. MNE estimates were averaged across different runs after being coregistered in the same coordinate system. Regions of interest (ROIs) for the MEG analysis were selected as the global maxima of cortical activity at the peak of the first cortical response after the stimuli onset. These ROIs will be referred to from now on as MEG-defined ROIs. Each MEG-defined ROI consisted of 10 neighboring vertices surrounding the vertex with the global maximum activation at the peak of the first cortical response after the stimuli onset. The average size of MEG-defined ROIs was 0.792 ± 0.169 mm. The mean distance of the vertices defining the ROI from the central vertices was 3.4 ± 1.47 mm. Then, the exact location of each MEG-defined ROI was displayed on the anatomical MRI of each individual.

### MRI-defined ROIs

T1 and diffusion data were processed with Connectome Mapper (CMP) (Daducci et al., 2012) pipeline, which includes the use of several neuroimaging tools, such as the FreeSurfer^2^. The CMP-generated file ROI_HR_th.nii.gz was used to create volume files for thalamus, pre-central gyrus, and post-central gyrus using the mri_binarize command of FreeSurfer. All three volumes were checked and manually edited in FreeView to ensure correct segmentation. The three volumes and the T1 image were coregistered with the b0 image using 3D Slicer software^3^. The transformed volumes were then imported in TrackVis software^4^ as ROIs. They will be referred to from now on as MRI-defined ROIs.

### Fiber tractography

Diffusion data were processed with Diffusion Toolkit^5^ using HARDI/Q-Ball imaging model and second order Runge Kutta propagation algorithm with an angle threshold of 45° and no FA threshold. Fiber tractography was performed with TrackVis software to create fiber tracks that pass through thalamus and post-central gyrus as well as thalamus and pre-central gyrus. Some spurious connections (<3%) were manually removed using TrackVis. Mean number of fibers as well as mean scalar measures of FA, ADC, axial diffusivity (AD), and radial diffusivity (RD) were derived for each fiber track. Data were analyzed separately for the two hemispheres identified as affected (or more affected) and intact (or less affected) hemispheres in CP children, and for right and left hemispheres for TD children.

### Resting-state fMRI

For the rs-fMRI analysis, we followed the same methodology as in Dehaes et al. (2013). Anatomical T1-weighted DICOM images were converted into a 3D NIFTI file format using the *mri_convert* command of FreeSurfer^2^. Then, the T1 image of each participant was manually oriented into Talairach space using the anterior commissure (AC) and posterior commissure (PC) landmarks using FreeView. Brain extraction tool (BET) was used to remove the non-brain tissue from the T1 image (Smith, 2002). Artifact detection for rs-fMRI raw data was performed using Artifact Rejection Tools (ART)^6^. All participants, except for one child with HCP who had three volumes affected by motion, were free of motion artifacts. After the removal of contaminated volumes, the remaining time series were converted into a 4D NIFTI volume file using *mri_convert* command of FreeSurfer. Slice timing correction was applied to the rs-fMRI data to correct for sampling offsets using the *slicetimer* command of FSL. The regression of motion signal was achieved using the *mcflirt* command of FSL (Jenkinson et al., 2002). Then, the rs-fMRI data were registered in the brain extracted structural T1-weighted image using the FSL’s intensity-based affine registration tool, *flirt*^7^ (Jenkinson et al., 2002). Before the statistical analysis, signal from cerebrospinal fluid (CSF) and white matter was regressed out.

For the statistical analysis, we used the Multivariate Exploratory Linear Optimized Decomposition into Independent Components (*melodic*) command of the FSL software, which allowed us to decompose the 4D rs-fMRI data sets into its spatial and temporal components with the use of ICA, which can be used to identify distinct RSNs (Beckmann and Smith, 2004). Number of components was not specified *a priori*. In this study, we have identified a sensorimotor RSN of all participants.

## Results

### Clinical data and MRI

Table 1 describes the type of lesions in the HCP and DCP children. No gross MRI abnormality was observed in one DCP child. All CP participants had visible asymmetry on the FA maps on the corticospinal tracts. HCP1 had severe spastic paresis of the upper limb and moderately impaired tactile sensory function and proprioception. HCP3, DCP1, and DCP4 had mild paresis of the hand and normal sensory function. The rest of CP participants had no evidence of motor or sensory impairment.

### Distributed source analysis

**Figure 1:**
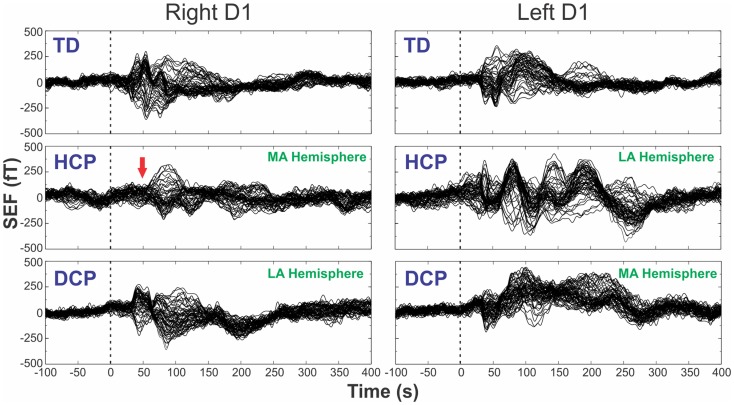
**SEFs evoked by the tactile stimulation of the right (left panels) and left (right panels) D1 from TD2, HCP1, and DCP3 children from −100 to 400 ms after the stimulus onset**. Red arrow indicates the absence of the first cortical response in the HCP1 participant.

Figure 1 shows the superimposed (butterfly) plots of the SEFs for a TD, an HCP, and a DCP child evoked by the tactile stimulation of right D1 and left D1, respectively. A weak reflection at ~30 ms was observed as the first cortical response after the stimulus onset, but only in four participants (two TD, one HCP, and one DCP) and only after few digits’ stimulation: TD1: left hand D1 at 28 ms, D3 at 27 ms; TD2: right hand D1 at 32 ms, left hand D1 at 28 ms and D5 at 27 ms; HCP1: left hand D1 at 28 ms, D3 at 30 ms, D5 at 31 ms; DCP2: right hand D1 at 29 ms. The most prominent early deflection evoked by the tactile stimulation of D1, D3, and D5 of both hands was observed at around 40–50 ms (M50) in all TD (mean latency ± SD for D1: 42.62 ± 3.55 ms, D3: 43.82 ± 3.88 ms, and D5: 44.24 ± 4.6 ms) and all DCP children (mean latency ± SD for D1: 43.36 ± 4.1 ms, D3: 45.03 ± 4.5 ms, and D5: 45.06 ± 2.0 ms). For the lowest functioning HCP child (HCP1), the M50 was absent in the SEFs elicited from the stimulation of the D1, D3, and D5 of the paretic hand (see Figure 1 for D1), while components at later latencies were present. Tactile stimulation of the non-paretic hand in the HCP children indicated altered SEFs in the later latencies. The M50 was present in the other two HCP children in both hemispheres. Isofield maps determined that the equivalent current dipoles (ECDs) for the components labeled as M30 presented an anterior ECD direction, while ECDs for the M50 components presented a posterior ECD direction.

**Figure 2:**
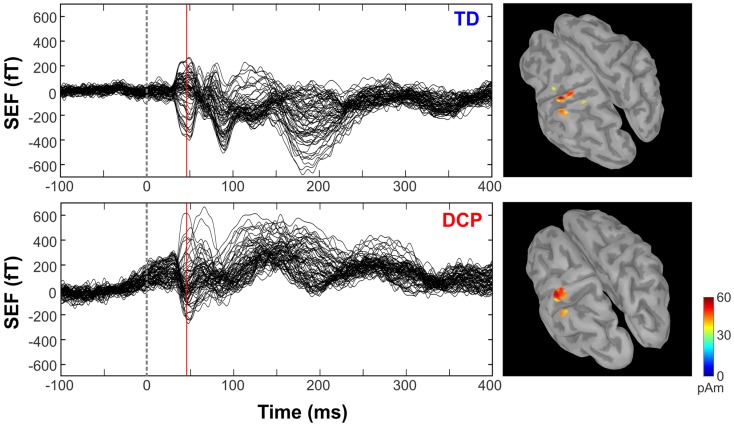
**Somatosensory-evoked fields evoked by the tactile stimulation of the right D1 (left panels) for TD1 and DCP2 (left hemisphere more affected) children respectively from −100 to 400 ms**. MNEs (in pico ampere meter) overlaid on participant cortical surfaces at the peak of M50.

**Figure 3:**
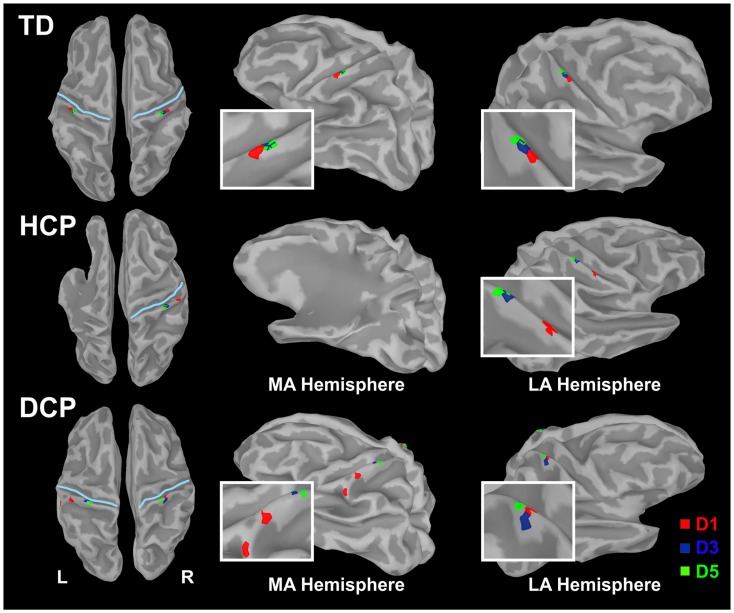
**Cortical responses in S1 at the peak of M50 for the tactile stimulation of D1, D3, and D5 of the right (left panel) and left (right panel) hands in the three representative participants (TD1, HCP1, and DCP2)**. Cortical responses were defined by the maxima MNEs elicited in the contralateral hemisphere. MA hemisphere, more affected hemisphere; LA, less affected hemisphere.

Figure 2 shows the butterfly plots of the SEFs for TD1 (upper panel) and DCP2 (lower panel) child accompanied by the corresponding MNE solutions at the peak of M50. At this latency, distributed activations were also observed in the post-central and pre-central gyri and the parietal lobe contralateral to the stimuli for all TD and DCP children. Figure 3 presents the M50 current sources (defined by maximal activities in the MNE maps) at the contralateral primary SS cortex (S1) for the stimulation of D1, D3, and D5 (both hands) for three representative participants from each group. Source analysis focused on the M50 that provided sufficient signal-to-noise (SNR) for reliable localization of the underlying generator. The M50 sources contralateral to the stimuli were located in the S1 area of the hand following a somatotopic order for both hands in all TD children (see Figure 3 – upper panel for TD1), in all HCP children for the non-paretic hand (see Figure 3 – middle panel – third column for HCP1), and in two DCP for the less paretic hand (see Figure 3 – lower panel – third column for DCP2): D5 medial and superior to D3, and D3 medial and superior to D1. An altered somatotopy was observed for the more paretic hand in two DCP children, and in the non-paretic hand in one HCP child (HCP2): D3 was located more posterior and inferior to D1 (Figure 3 – lower panel – second column for DCP2). Euclidean distances were markedly different among evoked activities in S1 between the TD group [mean distance ± SD (both hemispheres): for D1–D3: 7.38 ± 1.95 mm, for D3–D5: 6.89 ± 5.18 mm, for D1–D5: 8.18 ± 5.54 mm] and the HCP group for the more affected hand (mean distance ± SD: for D1–D3: 0.0 ± 0.00 mm, for D3–D5: 0.00 ± 0.00 mm, for D1–D5: 0.00 ± 0.00 mm) and the less affected hand (mean distance ± SD: for D1–D3: 12.95 ± 5.05 mm, for D3–D5: 10.58 ± 11.77 mm, for D1–D5: 21.47 ± 9.44 mm); Euclidean distances were markedly larger in the TD group compared to the HCP group for the more affected hemisphere and markedly shorter in the TD compared to the HCP for the less affected hemisphere. In the DCP children, Euclidean distances of S1 activities were markedly larger for both hemispheres compared to both hemispheres of the TD children. The Euclidean distances between activities within the contralateral S1 due to stimulation of D1, D3, and D5 for all participants are presented in Table 2.

**Table 2:** **Euclidean distances (in millimeter) for activities in S1**.

Participant	D1–D3		D3–D5		D1–D5	
	RH	LH	RH	LH	RH	LH
TD1	7.79	7.82	7.79	14.85	0.00	7.56
TD2	9.42	3.72	10.50	1.00	14.77	3.72
TD3	7.13	8.44	4.35	2.87	12.11	10.96
Average	8.11	6.66	7.54	6.24	8.96	7.41

	**MA**	**LA**	**MA**	**LA**	**MA**	**LA**

HCP1	*	18.41	*	5.88	*	24.23
HCP2	0.00	8.44	0.00	23.97	0.00	29.24
HCP3	0.00	12.00	0.00	1.88	0.00	10.96
Average	0.00	12.95	0.00	10.58	0.00	21.47

	**MA**	**LA**	**MA**	**LA**	**MA**	**LA**

DCP1	5.15	–	–	–	–	–
DCP2	20.63	11.31	28.03	7.54	10.29	11.55
DCP3	8.34	31.49	9.94	6.54	16.44	25.17
DCP4	33.82	10.12	9.71	11.16	38.36	18.98
Average	16.98	17.64	15.89	8.41	21.69	18.56

*, Absent activity.

*–, No data*.

### Tractography results

**Figure 4:**
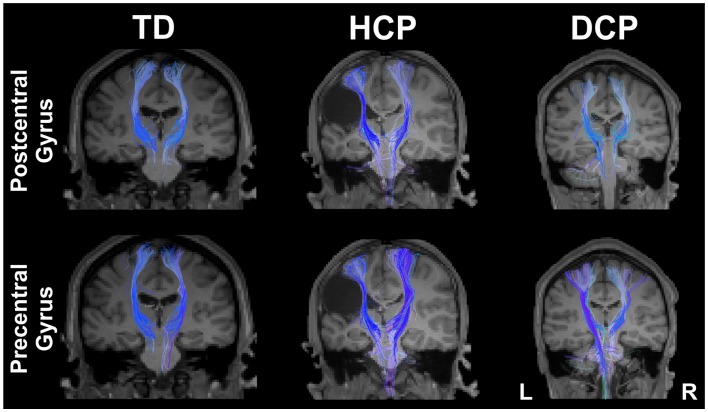
**Thalamocortical fibers projecting from thalamus to the post-central gyrus (upper panel) and from thalamus to pre-central gyrus (lower panel) for both sides of the brain for the same participants as in Figure 1 (TD2, HCP1, and DCP3 children)**.

**Figure 5:**
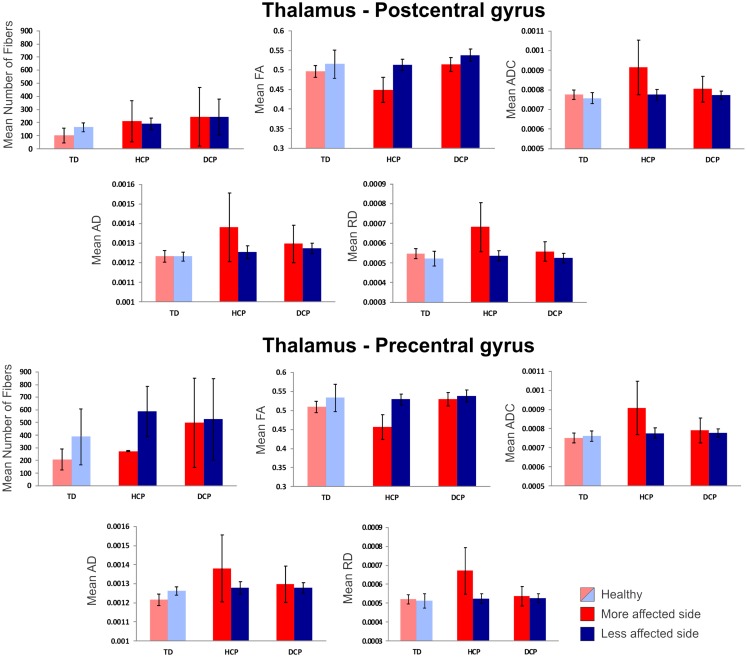
**Mean diffusivity values (±SD) for the thalamocortical fibers projecting from thalamus to post-central gyrus (upper panel) and thalamus to pre-central gyrus (lower panel) for all participants**. Red indicates the affected (or more affected) hemisphere; blue indicates the intact (or less affected) hemisphere. Light red/blue indicate left and right hemisphere in TD.

Figure 4 presents the thalamocortical tracks from thalamus to post-central gyrus (upper panel) and thalamus to pre-central gyrus (lower panel) for the same individuals as in Figure 1. The number of fibers between thalamus and post-central gyrus was not noticeably different between the two hemispheres in any of the participant groups (Figure 5 – upper panel). Mean FA values for the thalamocortical fibers from thalamus to post-central gyrus were lower for the affected compared to the non-affected hemisphere in both CP groups and this difference was clearly visible in the HCP group (Figure 5 – upper panel). Mean ADC, AD, and RD values for the thalamocortical fibers from thalamus to post-central gyrus were markedly higher for the affected than the non-affected hemisphere in both CP groups, where the difference was more pronounced in the HCP group (Figure 5 – upper panel).

The mean number of fibers between thalamus and pre-central gyrus was higher in the non-affected hemisphere compared to the affected hemisphere in both CP groups and this difference was markedly larger in the HCP group (Figure 5 – lower panel). Mean FA values for the fibers between thalamus and pre-central gyrus were lower for the affected compared to the non-affected hemisphere in both CP groups, where the difference was most visible in the HCP group (Figure 5 – lower panel). Moreover, mean ADC, AD, and RD values of the thalamocortical fibers from thalamus to pre-central gyrus were higher for the affected than the non-affected hemisphere in both CP groups and the differences were always more pronounced in the HCP group (Figure 5 – lower panel).

Mean diffusivity values for each individual in the three participant groups mostly followed the trend that we observed in the group averages for both fiber tracts (thalamus to post-central gyrus and thalamus to pre-central gyrus). Individual results are presented in Tables 3 and 4. The most striking result belonged to the HCP child (HCP1) with the large cortical lesion on the left hemisphere. For both fiber tracts, the mean ADC, AD, and RD values for this child’s affected hemisphere was markedly increased compared to the non-affected hemisphere, while the mean FA values for the affected hemisphere was notably decreased than the non-affected hemisphere. Another interesting finding was of a DCP child (DCP3), whose left leg was affected and had a normal MRI of the brain according to the radiology report. Due to the left foot paresis, this child’s right hemisphere was considered to be more affected; however, the results were not straightforward. For thalamus to post-central gyrus fiber tracts, this child’s affected hemisphere did indeed had a lower mean FA value than the non-affected hemisphere like the rest of the CP children, however, for thalamus to pre-central gyrus fiber tracts, mean FA value was markedly higher for the affected hemisphere than the non-affected hemisphere unlike the rest of the CP children. The mean ADC, AD, and RD values for this child’s affected hemisphere were lower compared to the non-affected hemisphere for both fiber tracts. Nevertheless, the differences between the mean diffusivity values of the two hemispheres in DCP3 were not too large.

**Table 3:** **DTI results from thalamus to post-central gyrus (mean values for the more affected and less affected hemispheres)^a^**.

Participant	NoF		FA		ADC		AD		RD	
	MA	LA	MA	LA	MA	LA	MA	LA	MA	LA
TD1	53	170	0.4798	0.4816	0.000804	0.000773	0.001262	0.001216	0.000575	0.000552
TD2	84	193	0.5101	0.5529	0.000757	0.000727	0.001203	0.001221	0.000535	0.000479
TD3	161	127	0.4969	0.5099	0.000765	0.000774	0.001231	0.001257	0.000531	0.000532
Average	99.3	163	0.4956	0.5148	0.000775	0.000758	0.001232	0.001231	0.000547	0.000521
HCP1	262	136	0.4142	0.5184	0.001075	0.000750	0.001582	0.001219	0.000821	0.000516
HCP2	30	215	0.4545	0.5233	0.000849	0.000771	0.001274	0.001260	0.000636	0.000526
HCP3	334	215	0.4789	0.4991	0.000818	0.000801	0.001285	0.001282	0.000692	0.000560
Average	209	189	0.4492	0.5136	0.000914	0.000774	0.001380	0.001254	0.000716	0.000534
DCP1	491	387	0.5169	0.5201	0.000850	0.000797	0.001367	0.001290	0.000592	0.000551
DCP2	52	110	0.4951	0.5505	0.000833	0.000768	0.001334	0.001288	0.000582	0.000508
DCP3	188	227	0.5299	0.5412	0.000729	0.000754	0.001188	0.001241	0.000499	0.000510
Average	244	241	0.5140	0.5373	0.000804	0.000773	0.001296	0.001273	0.000558	0.000523

*^a^NoF, number of fibers; FA, fractional anisotropy; ADC, apparent diffusion coefficient; AD, axial diffusivity; RD, radial diffusivity; MA, more affected hemisphere; LA, less affected hemisphere*.

**Table 4:** **DTI results from thalamus to pre-central gyrus (mean values for the more affected and less affected hemispheres)^a^**.

Participant	NoF		FA		ADC		AD		RD	
	MA	LA	MA	LA	MA	LA	MA	LA	MA	LA
TD1	135	188	0.4743	0.4946	0.000784	0.000777	0.001225	0.001239	0.000563	0.000546
TD2	187	627	0.5317	0.5699	0.000718	0.000731	0.001182	0.001250	0.000486	0.000471
TD3	298	345	0.5212	0.5347	0.000752	0.000777	0.001239	0.001296	0.000508	0.000518
Average	207	387	0.5091	0.5331	0.000751	0.000762	0.001215	0.001262	0.000519	0.000512
HCP1	273	533	0.3972	0.5283	0.001105	0.000779	0.001603	0.001278	0.000857	0.000530
HCP2	280	809	0.4864	0.5635	0.000814	0.000745	0.001276	0.001274	0.000584	0.000480
HCP3	271	421	0.4864	0.4949	0.000805	0.000803	0.001263	0.001284	0.000574	0.000560
Average	275	588	0.4567	0.5289	0.000908	0.000776	0.001381	0.001279	0.000672	0.000523
DCP1	848	829	0.4976	0.5304	0.000840	0.000788	0.001322	0.001295	0.000599	0.000535
DCP2	507	558	0.5506	0.5667	0.000786	0.000747	0.001344	0.001281	0.000507	0.000480
DCP3	141	188	0.5390	0.5168	0.000743	0.000796	0.001224	0.001257	0.000503	0.000565
Average	499	525	0.5291	0.5380	0.000790	0.000777	0.001297	0.001278	0.000536	0.000527

*^a^NoF, number of fibers; FA, fractional anisotropy; ADC, apparent diffusion coefficient; AD, axial diffusivity; RD, radial diffusivity; MA, more affected hemisphere; LA, less affected hemisphere*.

### rs-fMRI results

The number of spatially independent component maps ranged from 76 to 98 for the nine participants. We identified four ICA maps representing two anatomically distinct RSNs associated with the sensorimotor area. RSN1 and RSN2 were unilateral SS networks in the right or the left hemisphere, and RSN3 was a bilateral SS network. Both of these networks included activation in pre-central and post-central gyri, which are areas that represent the hand region in S1 and M1 in TD individuals (Liu et al., 2008). RSN4 was a motor network that included activation in the midline and its surrounding areas, which are shown to represent the foot and leg region in TD individuals (Luft et al., 2002; Kapreli et al., 2006; Christensen et al., 2007). Similar patterns have been identified in previous RSN studies (Biswal et al., 1995; Beckmann et al., 2005; Liu et al., 2008). Table 5 shows the RSNs identified for each participant.

**Table 5:** **Somatosensory (SS) and motor RSNs**.

Participant	Affected side	Components	SS right	SS left	SS bilateral	Motor
TD1	N/A	80	+	+	Symmetrical	+
TD2	N/A	76	+	+	Symmetrical	+
TD3	N/A	98	+	+	Symmetrical	+
HCP1	Right	81	+	−	−	+
HCP2	Left	89	−	−	Asymmetrical (left larger)	+
HCP3	Right	91	+	+	Asymmetrical (right larger)	+
DCP1	Both (right more)	94	−	−	Symmetrical	+
DCP2	Both (right more)	88	−	+	Asymmetrical (left larger)	+
DCP3	Both (left more)	79	−	−	Symmetrical	+

All three SS ICA maps were present for each TD participant. Moreover, the bilateral SS network had a symmetrical pattern in all TD children. The most affected HCP participants did not have all of the three SS RSNs. HCP1, who had a large lesion in his left hemisphere did not have the left sided and the bilateral SS RSNs. HCP2 whose right hemisphere was affected, did not have separate SS RSNs for the right and left hemisphere and had an asymmetrical bilateral SS RSN where the left side activation was more widespread. HCP3 was the least affected child with HCP whose left hemisphere was affected. For him, we could find all the three SS RSNs. Nevertheless, his bilateral SS RSN was asymmetrical where the right hemisphere had a more widespread activity. We observed the fewest number of SS RSNs for the participants with DCP. DCP1 did not have either of the right and left SS RSNs, while her bilateral SS RSN was symmetrical. DCP2 did not have a right-sided SS RSN and her bilateral SS activity was larger on the left hemisphere. DCP3 did not have either of the right and left SS RSNs, and his bilateral SS RSN had symmetrical activity on both hemispheres.

The motor RSN was present in all nine participants. Within-group average results show that the localization of activity in the TD and HCP groups was similar and more restricted to the midline. However, the activity was stronger in the HCP group than in the TD group. On the other hand, the activity was more widespread in the DCP group than the other two participant groups, also covering the primary and secondary SS regions other than the motor region, a finding, which is similar to the one found in Burton et al. (2009).

## Discussion

Our preliminary multimodal neuroimaging findings suggest an abnormal SS processing mechanism in children with SCP. This mechanism could be the result of a possible reorganization process. Our results evident an altered SS mechanism as: (i) an altered morphology of the evoked responses elicited contralateral to the stimuli in both hemispheres of DCP and HCP children; (ii) an altered somatotopic representation of the hand areas in the contralateral S1 to the more paretic hand of two DCP children and to the non-paretic hand of one HCP child, (iii) markedly different Euclidean distances between S1 activities elicited by tactile stimulation of D1, D3, and D5 between the two hemispheres in the HCP children and between DCP and TD children for both hemispheres, and (iv) absent and/or abnormal sensorimotor RSNs for children with HCP and DCP. Functional abnormalities detected by MEG and rs-fMRI in CP children were supported by our DTI findings, which indicate structural deficits in the thalamocortical fibers projecting from thalamus to the pre-central and post-central gyri.

Functional alterations in the magnetic evoked responses observed here have also been previously reported after the tactile stimulation of fingers and after the electrical stimulation of the median nerve in HCP children with subcortical lesions (Nevalainen et al., 2012). These alterations include missing deflections, aberrant morphology, and different distances in the localized SEFs components between CP and TD patients. Such findings could be due to diminished thalamocortical projections from thalamus to S1 (Hoon et al., 2002; Lee et al., 2005) and/or subsequent aberrant functioning of cortical SS network. Our Euclidean distance results are in line with Nevalainen et al. (2012) that reported significant different distances between the representation areas of D1, D3, and D5 in the more affected hemisphere of HCP children compared to TD. Our most striking MEG finding was the altered somatotopy in the hemisphere contralateral to the most paretic hand in two DCP children and to the non-paretic hand in one HCP child. To our best knowledge, it is the first time that an altered somatotopic order is reported in CP.

In contrast with previous EEG and MEG studies (Teflioudi et al., 2011; Guo et al., 2012; Pihko et al., 2014), but in line with others (Nevalainen et al., 2012), we did not observe differences in latencies of the first prominent cortical response at M50 between the CP and TD children. Alterations in later deflections that were observed bilaterally in the DCP children likely reflect aberrant processing in the local cortical network after the arrival of the thalamocortical input to the cortex. Guo et al. (2012) reported delayed latencies for the first SEFs response in CP compared to TD children. Our group also observed similar findings in an adult CP patient with a large unilateral, prenatally acquired, periventricular brain lesion (Papadelis et al., 2012). On the other hand, other studies have reported the first cortical response at normal latencies after tactile stimulation of the paretic thumb of CP children with PVL, localized in its original topography in the Rolandic region of the affected hemisphere (Gerloff et al., 2006; Staudt et al., 2006; Wilke and Staudt, 2009). The contradictory findings between all these studies might be explained by the possible different timing of the insults in these patients. The maturational stage of the brain at the time of the insult determines the type of structural pathology and eventually the possible reorganization mechanism (Staudt, 2007).

One of our HCP children (HCP1) had a large lesion on his left hemisphere due to prenatal left middle cerebral artery infarct. For this child, the M50 component was absent from the SEFs in the contralateral hemisphere when the digits of the paretic hand were stimulated. Missing early cortical responses have been previously reported by Pihko et al. (2014) after the stimulation of the median nerve in CP children. There are two possible mechanisms that can explain our finding for the HCP1: (i) the primary SS representation of the paretic hand has been transferred to the contralesional hemisphere, ipsilateral to the stimulation due to damaged projections of the afferent thalamocortical fibers to S1, or (ii) the afferent thalamocortical SS projections had apparently “bypassed” the lesion and reached their original cortical destination area in the post-central gyrus, but the bypass caused a significant delay in the first cortical response that appeared later in time (see Figure 1 – left middle panel). Unfortunately, the partial coverage of our system’s sensor array does not allow us to conclude which mechanism was present but only to speculate. A previous study by Staudt et al. (2006) in hemiparetic patients has supported the later explanation as the most possible one. It was found that despite the large periventricular lesions of their patients, the S1 representation of the paretic hand was located in the Rolandic cortex of their affected hemisphere. They assumed that the outgrowing thalamocortical projections might have developed as a consequence of compensation after the insult. In our HCP1 patient, these axons may not have found their way to the preserved tissue around the periventricular defect and may project to the ipsilateral hemisphere. Although this is a plausible explanation, the exact timing of the insult remains unclear in all these studies. A later cortical response peaking at ~80 ms was observed in this child that was localized in the pre-central gyrus. This response was prolonged looking similar to components that occur at later latencies (~80–100 ms after stimulus onset) in the normal SS processing and probably reflects secondary processes in the SS cortex. The localization of this component in the pre-central gyrus might be explained by the missing secondary SS cortex in the lesioned hemisphere. Such a later component was also present in the TD and DCP children at the same latency (see Figure 1).

We further used two neuroimaging methods, namely DTI and rs-fMRI, that can help us disentangle this issue. Several previous studies have used DTI to investigate microstructural abnormalities responsible for motor weakness and disability in children with CP because conventional MRI is unable to detect subtle structural abnormalities (Lee et al., 2003). However, to our knowledge, no study yet reported the integrity of motor and sensory white matter pathway differences among TD, HCP, and DCP children. In our study, we found decreased FA values for the thalamocortical pathways in the affected (compared to the unaffected) hemisphere in children with CP, which may reflect a loss or disorganization of the structural barriers to molecular diffusion of water in these patients (Trivedi et al., 2010; Rai et al., 2013). Increased ADC values could be suggestive of increased extracellular water content due to gliosis and microscopic cystic changes emerging at the affected brain regions in CP children (Trivedi et al., 2010; Rai et al., 2013). In our findings, the increase in ADC values was accompanied by an increase both in AD and, primarily, RD. Studies have shown that while an increase in RD might be a sign of disorganized, demyelinated, dysmyelinated, and/or poorly myelinated axons (Song et al., 2002, 2005; Nair et al., 2005), an increase in AD is associated with axonal injury or damage, which causes a decrease in axonal density or caliber, finally resulting in an increase in the extra-axonal space allowing water molecules to move faster (Song et al., 2005; Sun et al., 2008; Kumar et al., 2010; Della Nave et al., 2011).

We found that children with HCP showed the most noticeable FA and ADC value differences between their affected and non-affected hemispheres for both of the pathways that we studied. The mean number of tracts was considerably higher for the non-affected hemisphere of HCP children compared to their affected hemisphere and to the healthy hemispheres of TD children. Although this change does not necessarily directly translate into an increase in the actual number of axons (Koerte et al., 2011), it may indicate a possible reorganization of the thalamus to the pre-central gyrus. Our finding is in parallel with previous studies, which showed that quadriplegic CP children were more affected (decreased mean FA and increased mean ADC in corticospinal tract) than DCP (Chang et al., 2012), and CP children with low gross motor function were more affected (lower number of fibers in corticospinal tract) than ones with high gross motor function (Rha et al., 2012).

Our DTI results indicate anatomical deficits in both the quality and quantity of thalamocortical fibers projecting from thalamus to the pre-central and post-central gyri. The diminished quality of the ascending thalamocortical fibers projecting from thalamus to the post-central gyrus may explain the observed abnormalities in the SEFs. Our DTI findings also support the notion that anatomical deficits in the sensory pathways, in addition to the motor pathways, could be responsible for the pathophysiology of motor disability and weakness in children with CP. By extending the findings of previous studies, we show a clear difference in the sensory and motor pathways of children with HCP compared to DCP and TD children, as well as an indication of possible reorganization of the tracks from thalamus to pre-central gyrus in HCP children.

**Figure 6:**
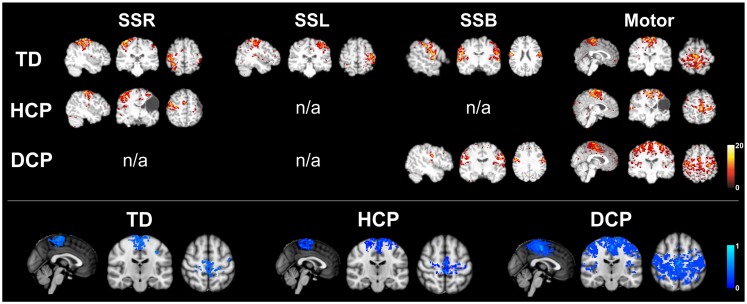
**Upper panel: RSNs for the same participants as in Figure 1 (TD2, HCP1, and DCP3 children)**. SSR, right somatosensory RSN; SSL, left somatosensory RSN; SSB, bilateral somatosensory RSN; motor, motor RSN (*z*-scores). Lower panel: mean motor RSN (lower panel) for the three groups of participants.

In order to test the functionality of the cortical SS network in CP children, we have also examined the sensorimotor RSNs in the three groups of children using an ICA method. For TD children, we managed to identify all the known SS and motor RSNs. HCP and DCP children showed absent and/or abnormal sensorimotor RSNs consistent with the severity and location of their lesions. Participants with HCP had absent or weak SS networks on their lesioned hemispheres while the intact hemisphere seemed to have stronger and more widespread activation (see Figure 6). Participants with DCP showed widespread activity for the motor RSN, extending to the SS cortices compared to the other participant groups, which is a similar finding as in Burton et al. (2009). The more widespread and/or stronger activity in participants with CP could be explained by disruptions to the somatotopic organizations in the SS cortices and the development of new and the strengthening of already existing intracortical connections (Marin-Padilla, 1997; Burton et al., 2009). The findings are in parallel with our DTI findings where the number of fibers for DCP children was low for the thalamus and post-central pathway but high for thalamus to pre-central pathway for both hemispheres.

### Methodological limitations

Magnetoencephalography findings were used to determine the S1 functionally for the DTI analysis. In all CP and TD children, S1 activity was localized within the post-central gyrus. Ideally, the MEG-defined ROIs should be used for the determination of ROIs in the DTI analysis. However, fiber tracking has limitations in determining the thalamocortical SS radiations and the motor tracts for the hand areas due to its well-known weakness to handle crossing fibers at the centrum semiovale level (Nowinski et al., 2012). Here, we were not able to reconstruct fibers passing from the MEG-defined ROIs to the thalamus. Therefore, for the DTI analysis, we considered the volume of the entire post-central gyrus as the ROI. Since our experimental setup did not involve any spontaneous movements from the participants, we were unable to determine the M1 functionally. Although MNE indicated cortical activity within the pre-central gyrus, this activity was not considered to be significant functionally. There is evidence that MNE solutions of even focal sources can extend across sulcal walls separated by only a few millimeters (Liu et al., 2002; Lin et al., 2006b; Hauk et al., 2011). The observed activity within the pre-central gyrus was probably due to the expansion of MNE solutions from the actual primary generator located within the post-central gyrus to more anterior locations. Since we were unable to functionally define the M1 by using our MEG data, we followed the most common practice to define it anatomically by choosing the volume of the entire pre-central gyrus as the ROI (Trivedi et al., 2010; Chang et al., 2012; Rha et al., 2012).

## Conclusion

Even though our study is limited by the relatively small number of participants and the lack of statistical results, it is the first study that reports findings from multiple neuroimaging modalities in the same CP patients. Our functional and anatomical findings provide preliminary evidence of impaired SS processing in CP patients that are due to (i) diminished thalamocortical projections from thalamus to S1 (Hoon et al., 2002; Lee et al., 2005), and (ii) subsequent aberrant functioning of the cortical SS network. It should be noted though that our findings are limited due to the partial head coverage of our BabySQUID MEG system that does not allow simultaneous recordings of both hemispheres. Also, our small sample size prevents us from generalizing our conclusions to the general CP population. We conclude that motor and sensory pathways could both be important in the clinical outcome of children with CP and that the diminished connectivity in these pathways in the affected hemisphere, especially of children with HCP, could be indicative of a pathophysiological mechanism responsible for motor dysfunction (Yoshida et al., 2010; Lee et al., 2011). These results have important implications for the diagnosis and the rehabilitation of patients with CP. Such interventions should be applied early in life when the brain demonstrates the ability to plasticity, allowing it to readily reorganize in the face of injury (Johnston, 2009). Therefore, new approaches using innovative technologies that will identify early functional and structural deficits in the brain of children with PVL are needed.

## Conflict of Interest Statement

The authors declare that the research was conducted in the absence of any commercial or financial relationships that could be construed as a potential conflict of interest.

## Footnotes

^1^http://neuroimage.usc.edu/brainstorm

^2^http://surfer.nmr.mgh.harvard.edu/

^3^http://www.slicer.org

^4^http://www.trackvis.org

^5^http://www.trackvis.org/dtk/

^6^http://www.nitrc.org/projects/artifact_detect

^7^www.fmrib.ox.ac.uk/fsl
